# Correction: A multi-proxy assessment of the impact of environmental instability on Late Holocene (4500–3800 BP) Native American villages of the Georgia coast

**DOI:** 10.1371/journal.pone.0307121

**Published:** 2024-07-10

**Authors:** Carey J. Garland, Victor D. Thompson, Matthew C. Sanger, Karen Y. Smith, C. Fred T. Andrus, Nathan R. Lawres, Katharine G. Napora, Carol E. Colaninno, J. Matthew Compton, Sharyn Jones, Carla S. Hadden, Alexander Cherkinsky, Thomas Maddox, Yi-Ting Deng, Isabelle H. Lulewicz, Lindsey Parsons

The fifth author’s name is spelled incorrectly. The correct name is: C. Fred T. Andrus. The correct citation is: Garland CJ, Thompson VD, Sanger MC, Smith KY, Andrus CFT, Lawres NR, et al. (2022) A multi-proxy assessment of the impact of environmental instability on Late Holocene (4500–3800 BP) Native American villages of the Georgia coast. PLOS ONE 17(3): e0258979. https://doi.org/10.1371/journal.pone.0258979
