# Supplementary material for: A multi-proxy assessment of the impact of environmental instability on Late Holocene (4500-3800 BP) Native American villages of the Georgia coast
Source: PLoS One. 2022 Mar 2;17(3):e0258979. doi: 10.1371/journal.pone.0258979 (PMC8890641; doi:10.1371/journal.pone.0258979)
Supplement: S1 Table — (DOCX) [file pone.0258979.s001.docx]

**Table S1**: Sapelo Shell Ring Complex, Ring III, Unit 9 Species List.

| **Taxa** | | **NISP** | **MNI** | **%** | **Weight, g** | **Biomass, kg** |
| --- | --- | --- | --- | --- | --- | --- |
| Myliobatiformes | Rays | 3 | — | — | 0.025 | 0.005 |
| Dasyatidae | Stingrays | 1 | 1 | 0.5 | 0.011 | 0.003 |
| Actinopterygii | Indeterminate bony fishes | 6540 | — | — | 57.343 | 0.784 |
| *Anguilla rostrata* | American eel | 2 | 1 | 0.5 | 0.010 | 0.001 |
| Clupeidae | Herrings and shads | 3 | 1 | 0.5 | 0.003 | 0.000 |
| Siluriformes | Catfishes | 1483 | — | — | 24.143 | 0.411 |
| Ariidae | Sea catfishes | 764 | — | — | 28.624 | 0.483 |
| *Ariopsis felis* | Hardhead catfish | 2115 | 66 | 36.3 | 121.270 | 1.904 |
| *Bagre marinus* | Gafftopsail catfish | 21 | 3 | 1.6 | 1.644 | 0.032 |
| *Opsanus* spp. | Toadfish | 9 | 2 | 1.1 | 0.656 | 0.021 |
| *Mugil* spp. | Mullet | 169 | 5 | 2.7 | 2.019 | 0.052 |
| Cyprinodontidae | Killifishes | 25 | 3 | 1.6 | 0.085 | 0.004 |
| *Orthopristis chrysoptera* | Pigfish | 5 | 1 | 0.5 | 0.041 | 0.002 |
| *Archosargus probatocephalus* | Sheepshead | 2 | 1 | 0.5 | 0.405 | 0.007 |
| Sciaenidae | Drums | 324 | — | — | 2.316 | 0.072 |
| *Bairdiella chrysoura* | Silver perch | 27 | 5 | 2.7 | 0.726 | 0.031 |
| *Cynoscion* spp. | Seatrout | 32 | 3 | 1.6 | 1.512 | 0.053 |
| *Leiostomus xanthurus* | Spot | 19 | 4 | 2.2 | 0.204 | 0.012 |
| *Menticirrhus* spp. | Kingfish | 2 | 1 | 0.5 | 0.107 | 0.007 |
| *Micropogonias undulatus* | Atlantic croaker | 24 | 5 | 2.7 | 1.591 | 0.053 |
| *Sciaenops ocellatus* | Red drum | 2 | 1 | 0.5 | 0.235 | 0.013 |
| *Stellifer lanceolatus* | Star drum | 512 | 68 | 37.4 | 9.638 | 0.208 |
| *Peprilus* spp. | Harvest and butterfish | 4 | 1 | 0.5 | 0.024 | 0.005 |
| *Prinotus* sp. | Searobin | 1 | 1 | 0.5 | 0.004 | 0.001 |
| Paralichthyidae | Sand flounders | 2 | — | — | 0.098 | 0.003 |
| *Citharichthys* spp. | Whiff | 5 | 1 | 0.5 | 0.023 | 0.001 |
| *Paralichthys* spp. | Southern flounder | 7 | 2 | 1.1 | 0.495 | 0.014 |
| *Rana* sp. | Bullfrog | 1 | 1 | 0.5 | 0.006 | — |
| *Alligator mississippiensis* | American alligator | 1 | 1 | 0.5 | 0.136 | 0.002 |
| Testudines | Indeterminate turtles | 9 | 1 | 0.5 | 1.285 | 0.037 |
| Aves | Indeterminate birds | 1 | 1 | 0.5 | 0.065 | 0.002 |
| Mammalia | Indeterminate mammals | 14 | — | — | 2.613 | 0.062 |
| Sigmodontinae | American mice and rats | 2 | 1 | 0.5 | 0.018 | 0.001 |
| *Procyon lotor* | Raccoon | 1 | 1 | 0.5 | 1.374 | 0.035 |
| Vertebrata | Indeterminate vertebrates | — | — | — | 3.610 | — |
| **Total** |  | 12132 | 182 | 100% | 262.359 | 4.321 |
