# Supplementary material for: A multi-proxy assessment of the impact of environmental instability on Late Holocene (4500-3800 BP) Native American villages of the Georgia coast
Source: PLoS One. 2022 Mar 2;17(3):e0258979. doi: 10.1371/journal.pone.0258979 (PMC8890641; doi:10.1371/journal.pone.0258979)
Supplement: S2 Table — (DOCX) [file pone.0258979.s002.docx]

**Table S2:** Sapelo Shell Ring Complex, Ring III, Unit 4 Species List.

| **Taxa** | | **NISP** | **MNI** | **%** | **Weight, g** | **Biomass, kg** |
| --- | --- | --- | --- | --- | --- | --- |
| Carcharhinidae | Requiem sharks | 1 | 1 | 0.3 | 0.240 | 0.037 |
| Dasyatidae | Stingrays | 1 | 1 | 0.3 | 0.085 | 0.015 |
| Actinopterygii | Indeterminate bony fishes | 1573 | — | — | 98.295 | 1.213 |
| *Anguilla rostrata* | American eel | 2 | 1 | 0.3 | 0.117 | 0.005 |
| Siluriformes | Catfishes | 484 | — | — | 32.731 | 0.549 |
| Ariidae | Sea catfishes | 730 | — | — | 76.487 | 1.229 |
| *Ariopsis felis* | Hardhead catfish | 4361 | 243 | 73.0 | 612.876 | 8.871 |
| *Bagre marinus* | Gafftopsail catfish | 438 | 15 | 4.5 | 98.423 | 1.561 |
| *Opsanus* spp. | Toadfish | 14 | 2 | 0.6 | 2.971 | 0.321 |
| *Mugil* spp. | Mullet | 200 | 9 | 2.7 | 13.470 | 1.178 |
| *Fundulus* sp. | Killifish | 1 | 1 | — | 0.016 | 0.004 |
| *Pomatomus saltatrix* | Bluefish | 3 | 1 | 0.3 | 0.116 | 0.020 |
| Carangidae | Jacks and pompanos | 1 | 1 | 0.3 | 0.052 | 0.003 |
| *Archosargus probatocephalus* | Sheepshead | 1 | 1 | 0.3 | 0.079 | 0.002 |
| Sciaenidae | Drums | 60 | — | — | 6.120 | 0.149 |
| *Bairdiella chrysoura* | Silver perch | 3 | 1 | 0.3 | 0.044 | 0.004 |
| *Cynoscion* spp. | Seatrout | 240 | 22 | 6.6 | 17.196 | 0.319 |
| *Leiostomus xanthurus* | Spot | 5 | 2 | 0.6 | 0.063 | 0.005 |
| *Menticirrhus* spp. | Kingfish | 3 | 1 | 0.3 | 0.244 | 0.014 |
| *Micropogonias undulatus* | Atlantic croaker | 14 | 5 | 1.5 | 0.970 | 0.038 |
| *Pogonias cromis* | Black drum | 1 | 1 | 0.3 | 0.197 | 0.012 |
| *Sciaenops ocellatus* | Red drum | 36 | 4 | 1.2 | 15.495 | 0.296 |
| *Stellifer lanceolatus* | Star drum | 38 | 4 | 1.2 | 0.653 | 0.028 |
| *Peprilus* sp. | Harvest and butterfish | 1 | 1 | 0.3 | 0.004 | 0.000 |
| *Paralichthys* spp. | Southern flounder | 67 | 4 | 1.2 | 14.579 | 0.286 |
| *Alligator mississippiensis* | American alligator | 15 | 1 | 0.3 | 17.880 | 0.258 |
| Testudines | Indeterminate turtles | 58 | — | — | 10.305 | 0.151 |
| Kinosternidae | Mud and musk turtles | 9 | — | — | 1.744 | 0.046 |
| *Kinosternon* spp. | Mud turtle | 17 | 2 | 0.6 | 3.795 | 0.077 |
| Emydidae | Pond turtles | 9 | — | — | 3.567 | 0.074 |
| *Malaclemys terrapin* | Diamondback terrapin | 2 | 1 | 0.3 | 2.094 | 0.052 |
| Aves | Indeterminate birds | 10 | — | — | 1.444 | 0.029 |
| Ardeidae | Herons | 1 | 1 | 0.3 | 0.080 | 0.002 |
| Rallidae | Coots and rails | 10 | 2 | 0.6 | 3.044 | 0.056 |
| Mammalia | Indeterminate mammals | 365 | — | — | 212.817 | 3.275 |
| *Didelphis virginiana* | Opossum | 2 | 1 | 0.3 | 1.213 | 0.031 |
| Soricidae | Shrews | 1 | 1 | 0.3 | 0.003 | 0.000 |
| Sigmodontinae | American mice and rats | 1 | 1 | 0.3 | 0.088 | 0.003 |
| *Procyon lotor* | Raccoon | 5 | 1 | 0.3 | 5.646 | 0.125 |
| *Odocoileus virginianus* | White-tailed deer | 33 | 1 | 0.3 | 127.699 | 2.068 |
| Vertebrata | Indeterminate vertebrates | — | — | — | 12.098 | — |
| **Total** |  | 8816 | 333 | 100% | 1395.040 | 22.406 |
