# Supplementary material for: A multi-proxy assessment of the impact of environmental instability on Late Holocene (4500-3800 BP) Native American villages of the Georgia coast
Source: PLoS One. 2022 Mar 2;17(3):e0258979. doi: 10.1371/journal.pone.0258979 (PMC8890641; doi:10.1371/journal.pone.0258979)
Supplement: S3 Table — See Table 1 for corrected and modeled dates. (DOCX) [file pone.0258979.s003.docx]

**Table S3:** Uncorrected AMS dates and context for each sample. See Table 1 for corrected and modeled dates.

| **SITE NAME** | **UGAMS#** | **MATERIAL** | **CONTEXT** | **δ^13^C, ‰** | **^14^C years (BP)** | **±** |
| --- | --- | --- | --- | --- | --- | --- |
| Sapelo Shell Ring I | 15084 | sooted sherd | Unit 1 Level 2 | -17.0 | 3610 | 50 |
| Sapelo Shell Ring I | 15085 | sooted sherd | Unit 1 Level 2 | -18.9 | 3730 | 60 |
| Sapelo Shell Ring I | 52182 | UID Wood Charcoal | Unit 4 Level 7 | -25.9 | 3750 | 20 |
| Sapelo Shell Ring I | 52183 | deer bone | Unit 4 Level 9 | -22.89 | 3780 | 20 |
| Sapelo Shell Ring I | 52184 | deer bone | Unit 4 Level 10 | -22.67 | 3670 | 20 |
| Sapelo Shell Ring I | 52185 | deer bone | Unit 4 Level 10 | -21.29 | 3830 | 20 |
| Sapelo Shell Ring I | 52186 | Pinus spp. (Pine) | Unit 4 Level 11 | -26.73 | 3840 | 20 |
| Sapelo Shell Ring I | 52187 | deer bone | Unit 4 Level 11 | -22.43 | 3810 | 20 |
| Sapelo Shell Ring I | 52188 | Pinus spp. (Pine) | Unit 4 Level 13 | -25.74 | 3820 | 20 |
| Sapelo Shell Ring I | 52189 | deer bone | Unit 4 Level 13 | -22.62 | 3820 | 20 |
| Sapelo Shell Ring I | 52190 | Pinus spp. (Pine) | Unit 4 Level 14 | -25.44 | 3810 | 20 |
| Sapelo Shell Ring I | 52191 | deer bone | Unit 4 Level 17 | -22.8 | 3790 | 20 |
| Sapelo Shell Ring II | 52175 | *Carya* spp. *(Hickory nut)* | Unit-A1-Level 3 | -26.33 | 3680 | 20 |
| Sapelo Shell Ring II | 42750 | Pinus spp. (Pine) | Unit 1, 37.5 cmbs | -25.75 | 3800 | 20 |
| Sapelo Shell Ring II | 42751 | Pinus spp. (Pine) | Unit 1, 47 cmbs | -26.97 | 3770 | 20 |
| Sapelo Shell Ring II | 42752 | Pinus spp. (Pine) | Unit 1, 70-75 cmbs | -26.09 | 3810 | 20 |
| Sapelo Shell Ring III | 15082 | UID Wood Charcoal | Unit 9, Level 4 | -27.5 | 3560 | 50 |
| Sapelo Shell Ring III | 15083 | UID Wood Charcoal | Unit 9, Level 7 | -25.5 | 3730 | 60 |
| Sapelo Shell Ring III | 15086 | UID Charcoal | Unit 11, Level 4 | -25.6 | 3730 | 50 |
| Sapelo Shell Ring III | 52174 | Pinus spp. (Pine) | Unit 9 Level 5 | -26.84 | 3770 | 20 |
| Sapelo Shell Ring III | 52177 | UID Wood Charcoal | Unit 9 Level 2 | -25.23 | 3590 | 20 |
| Sapelo Shell Ring III | 52178 | *Carya* spp. *(Hickory nut)* | Unit 9 Level 3 | -25.09 | 3620 | 20 |
| Sapelo Shell Ring III | 52179 | *Carya* spp. *(Hickory nut)* | Unit 9 Level 8 | -26.29 | 3660 | 20 |
| Sapelo Shell Ring III | 52180 | *Carya* spp. *(Hickory nut)* | Unit 9 Level 8 | -25.13 | 3690 | 20 |
| Sapelo Shell Ring III | 52181 | UID Charcoal | Unit 9 Level 9 | -26.59 | 3570 | 20 |
