# Supplementary material for: A multi-proxy assessment of the impact of environmental instability on Late Holocene (4500-3800 BP) Native American villages of the Georgia coast
Source: PLoS One. 2022 Mar 2;17(3):e0258979. doi: 10.1371/journal.pone.0258979 (PMC8890641; doi:10.1371/journal.pone.0258979)
Supplement: S1 Text — (DOCX) [file pone.0258979.s004.docx]

# Supplemental Text

***Vertebrate Analysis***

Two samples from Ring III were analyzed by Colaninno and Compton [1], providing an overview of subsistence practices and the environmental context for which people conducted these practices. Samples include vertebrate fauna from Ring III Unit 4 (1 x 1 m) and Unit 9 (1 x 2 m). The Unit 4 sample is from general level excavations that were recovered with 6.35 mm mesh screen, whereas the Unit 9 sample is a 25 x 25 cm column sample that was water screened through fine-screen mesh (1.59 mm).

Both samples resulted in large quantities of vertebrate remains with the Unit 4 sample containing 8,816 vertebrate specimens representing a minimum of 333 individuals from 31 taxa. The Unit 9 sample contains 12,132 specimens representing at least 182 individuals from 27 taxa. In sum, these two collections include 20,948 specimens from 39 vertebrate taxa (Tables S1 and S2). Individuals identified as sharks, rays, and bony fish common to estuarine and inshore habitats of the Georgia coast dominate both collections (96% of the Unit 4 collection and 97% of the Unit 9 collection). Few non-fish individuals are present in either collection and those that are represent animals common to the various habitats of the sea islands and their surrounding waters. Of the fish taxa present, two species, hardhead catfish (*Ariopsis felis*) and star drum (*Stellifer lanceolatus*), are most frequently identified. Both species are common to Georgia estuaries and tolerate wide temperature and salinity ranges.

***Radiocarbon Analysis***

Table S3 shows all raw AMS dates and the context for each sample. We also provide the Oxcal code for our Bayesian Chronological Models.

**Supplemental References**

1. Colaninno CE, Compton JM (2019) Integrating vertebrate and invertebrate seasonality data from Ring III of the Sapelo Island Shell Ring Complex (9MC23). *Journal of Island and Coastal* *Archaeology* 14(4):560-583
