# Supplementary material for: A multi-proxy assessment of the impact of environmental instability on Late Holocene (4500-3800 BP) Native American villages of the Georgia coast
Source: PLoS One. 2022 Mar 2;17(3):e0258979. doi: 10.1371/journal.pone.0258979 (PMC8890641; doi:10.1371/journal.pone.0258979)
Supplement: S1 File — (DOCX) [file pone.0258979.s005.docx]

**Oxcal Code:**

Plot()

{

Sequence()

{

Boundary("Start: Ring I");

Phase(Ring I)

{

Sequence("1")

{

R_Date("52191", 3790, 20);

R_Date("52190", 3810, 20);

Phase("1")

{

R_Date("52189", 3820, 20);

R_Date("52188", 3810, 20);

};

R_Date("52187", 3810, 20);

R_Date("52186", 3840, 20);

Phase("2")

{

R_Date("52185", 3830, 20);

R_Date("52184", 3670, 20)

{

Outlier();

};

};

R_Date("52183", 3780, 20);

R_Date("52182", 3750, 20);

};

Phase()

{

R_Date("15085", 3730, 60);

R_Date("15084", 3610, 60);

};

};

Boundary("End: Ring I");

};

Sequence()

{

Boundary("Start: Ring II");

Phase(Ring II)

{

Sequence("1")

{

R_Date("42752", 3810, 20);

R_Date("42751", 3770, 20);

R_Date("42750", 3800, 20);

};

Phase()

{

R_Date("52175", 3680, 20);

};

};

Boundary("End: Ring II");

};

Sequence()

{

Boundary("Start: Ring III");

Phase(Ring III)

{

Sequence("1")

{

R_Date("52181", 3570, 20)

{

Outlier();

};

R_Date("52180", 3690, 20);

R_Date("52179", 3660, 20);

R_Date("15083", 3730, 60);

R_Date("52174", 3770, 20)

{

Outlier();

};

R_Date("15082", 3560, 50)

{

Outlier();

};

R_Date("52178", 3620, 20);

R_Date("52177", 3590, 20);

};

Phase()

{

R_Date("15086", 3730, 50);

};

};

Boundary("End: Ring III");

};

Order("order")

{

Date("=Start: Ring I")
